# Management of Failed Bioprosthetic Aortic Valves: Mitigating Complications and Optimizing Outcomes

**DOI:** 10.1155/2022/9737245

**Published:** 2022-09-02

**Authors:** Elizabeth L. Norton, Alison F. Ward, Adam Greenbaum, Kendra J. Grubb

**Affiliations:** ^1^Division of Cardiothoracic Surgery, Emory University, Atlanta, USA; ^2^Division of Cardiology, Emory University, Atlanta, USA; ^3^Emory University Structural Heart and Valve Center, Atlanta, USA

## Abstract

The use of bioprosthetic prostheses during surgical aortic valve replacements has increased dramatically over the last two decades, accounting for over 85% of surgical implantations. Given limited long-term durability, there has been an increase in aortic valve reoperations and reinterventions. With the advent of new technologies, multiple treatment strategies are available to treat bioprosthetic valve failure, including valve-in-valve (ViV) transcatheter aortic valve replacement (TAVR). However, ViV TAVR has an increased risk of higher gradients and patient prosthesis mismatch (PPM) secondary to placing the new valve within the rigid frame of the prior valve, especially in patients with a small surgical bioprosthesis *in situ*. Bioprosthetic valve fracture allows for placement of a larger transcatheter valve, as well as a fully expanded transcatheter valve, decreasing postoperative gradients and the risk of PPM.

## 1. Introduction

Treatment of aortic valve pathology has evolved over the past decade with the advent of transcatheter aortic valve replacement (TAVR). In Europe, TAVR first received Conformitè Europèenne (CE) Mark approval in 2007, and the number of patients undergoing TAVR grew exponentially. In the United States (US), clinical trials began in 2007 and TAVR gained Food and Drug Administration (FDA) approval in 2011 for inoperable patients with severe aortic stenosis. Since then, surgical aortic valve replacements (SAVR) have decreased slightly as TAVR approval expanded to patients of all surgical risk profiles in 2019 [1]. However, overall aortic valve replacements, including TAVR and SAVR, have increased [2].

More than 85% of SAVRs are with bioprosthetic valves [3], but one of the major limitations is durability. Bioprosthetic valve dysfunction (BVD) can be categorized as either nonstructural valve deterioration (NSVD)--paravalvular regurgitation, patient-prosthetic mismatch (PPM), malposition, valve embolization, valve thrombosis, or endocarditis, or structural valve deterioration (SVD)--permanent intrinsic changes to the valve [4]. Valve durability is dependent on the valve manufacturer and type of prosthesis. SVD is an irreversible process resulting in hemodynamic and clinical changes similar to native valve aortic stenosis and regurgitation, eventually resulting in the need for reoperation. SVD definitions differ in the literature, leading to varying rates of reported valve failure. In most SAVR series, valve failure has been defined as a need for reintervention, but this is not a true “incidence of failure.” Patients can experience significant SVD without undergoing reoperation due to the underdiagnosis of SVD, minimalization of SVD severity, or patients not being considered surgical candidates [5].

**Figure 1:**
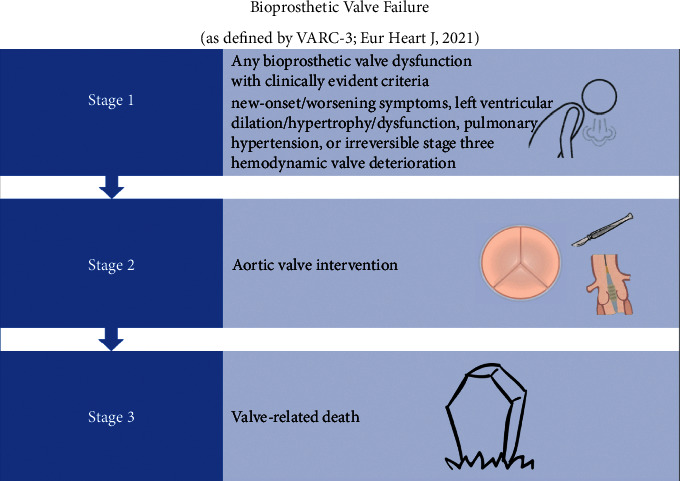
definition of Bioprosthetic Valve Failure. Adapted from VARC-3^*∗*^. ^*∗*^Varc-3 Writing C, Genereux P, Piazza N, et al. Valve Academic Research Consortium 3: Updated Endpoint definitions for Aortic Valve Clinical Research. J Am Coll Cardiol. 2021; 77 : 2717–2746.

The 2021 Valve Academic Research Consortium 3 (VARC-3) guidelines define bioprosthetic valve failure in three stages: (1) any bioprosthetic valve dysfunction with clinically expressive criteria (new-onset or worsening symptoms, left ventricular dilation/hypertrophy/dysfunction, pulmonary hypertension, or irreversible stage three hemodynamic valve deterioration), (2) aortic valve intervention, and (3) valve-related death [6] (Figure 1). With the increased use of bioprosthetic valves, an increase in reoperations or reinterventions for BVD is predicted. Management strategies continue to evolve and range from traditional redo-sternotomy SAVR, minimally invasive redo-SAVR, and placement of a TAVR valve in a failed SAVR, also known as valve-in-valve (ViV).

## 2. The Problem

### 2.1. Risk Factors for Bioprosthetic Valve Failure

Given the increase in bioprosthetic AVR utilization, the identification of predictors of valve failure and the recognition of opportunities to reduce the incidence of SVD are imperative. A variety of factors contribute to valve failure, including patient characteristics and comorbidities, type of implanted valve, and size of implanted valve. In a recent systematic review and meta-analysis, Ochi et al. identified multiple risk factors for BVD including younger age, sex, prosthesis brand, prosthesis size (<19 mm, <21 mm, <23 mm), PPM, absence of anticalcification preparation, concomitant coronary artery bypass graft surgery, subcoronary implantation technique, postoperative pressure gradient, dyslipidemia, smoking, metabolic syndrome, use of lipid lowering medication, elevated body mass index and body surface area, and renal disease. Meta-analysis identified younger age (per 1-year increase in age, HR = 0.91, *p* < 0.0001), increased body surface area (HR = 1.77, *p*=0.03), smoking (HR = 2.28, *p*=0.0015), and PPM (HR = 1.95, *p* < 0.0001) as the four significant determinants for SVD [7] (Table 1).

### 2.2. Patient-Prosthesis Mismatch

Patient-prosthesis mismatch occurs when the effective orifice area (EOA) of the implanted prosthetic valve is too small for the patient's body size [8]. PPM is defined by indexed EOA/body surface area (BSA) and is stratified by severity as follows: none (>0.85 cm^2^/m^2^), moderate (0.85 to 0.65 cm^2^/m^2^), and severe (≤0.65 cm^2^/m^2^). Fallon et al. reported that 65% of patients ≥65 years old with severe aortic stenosis who underwent SAVR had moderate or severe PPM [9]. Those patients with moderate or severe PPM had a significantly increased risk of readmission for heart failure (moderate, HR = 1.15, [95% CI: 1.09, 1.21]; severe, HR = 1.37, [95% CI: 1.26, 1.48]) and redo AVR (moderate, HR = 1.41, [95% CI: 1.13, 1.77]; severe, HR = 2.68, [95% CI; 2.01, 3.56]). Any degree of PPM has been associated with significantly lower survival [9, 10]. Older age, female sex, hypertension, diabetes, renal failure, larger BSA, and larger BMI have been identified as risk factors for PPM [11, 12]. TAVR has been associated with a decreased risk of PPM compared to SAVR, especially in patients with small aortic annuli. Aalaei-Andabili et al. found the incidence of PPM was almost double following SAVR compared to TAVR (54% vs. 29%, *p* < 0.001), especially among patients receiving a valve size ≤23 mm (SAVR, 65% vs. TAVR, 48%, *p*=0.048) [13]. The average aortic valve size implanted in the US is 22 mm [3], leaving many patients with the risk of PPM, early valve failure, and increased mortality. PPM can be mitigated at the time of initial AVR by implanting an appropriately sized valve.

### 2.3. Valve Sizing

When selecting a valve, the internal orifice diameter (ID) of the proposed implant should be identified, as the ID differs amongst valve models and manufacturers for the same labeled valve size. The largest valve that can be safely implanted is recommended, but strategies for selecting valve size differ in SAVR vs. TAVR. For SAVR, the valve size is selected by the surgeon at the time of implant based on manufacturer-specific annular valve sizers, while TAVR sizing relies entirely on preoperative computed tomography angiography (CTA). This difference in measurement results in valves with smaller ID being implanted during SAVR [14]. Preoperative CTA analysis defines the aortic annulus and root anatomy, allowing for an appropriately sized implant, SAVR or TAVR, to be selected. If a small aortic prosthesis is predicted, a root enlargement or root replacement can be performed at the time of SAVR. Alternatively, the initial valve that provides the largest EOA and best hemodynamics is often utilized at the time of TAVR. Many structural heart teams will assess every patient with CTA to ensure the most appropriately sized implant is utilized.

Especially in young patients with small annuli, an aortic root enlargement or replacement should be performed when the EOA index is ≤0.65 cm^2^/m^2^ and may be considered when the EOA index is ≤0.85 cm^2^/m^2^ [15]. Aortic root enlargement has not been widely adopted and is performed in <10% of SAVRs [3, 16]. Techniques for root enlargement include Nicks [17], Manouguian and Seybold-Epting [18], Konno et al. [19], and the Y-incision [20]. Both the Nicks and Manouguian procedures enlarge the aortic annulus via posterior extension of the aortotomy—the Nicks through the noncoronary sinus and the Manouguian through the left/noncoronary commissure, extending onto the anterior mitral leaflet, then closure with patch augmentation [17, 18]. The annular patch enables the implantation of a valve size 1–2 sizes larger than the native annulus [21]. A Konno, rarely done in adults, is an anterior annular augmentation extending onto the right ventricle [19]. The Y-incision, also a posterior enlargement, undermines the left and noncoronary cusps and enables implantation of a valve 2-3 sizes larger, with reports of up to 5 sizes larger [20, 22, 23]. A posterior aortic root enlargement is not associated with increased risk of mortality or adverse events at expert centers and can facilitate future ViV TAVR but absolutely precludes balloon fracture as the native annulus is unsupported [16, 24].

## 3. Solutions

Once clinically significant BVD occurs, intervention is indicated. Redo-SAVR may not be appropriate for all patients and a full imaging assessment with CTA and heart team discussion is necessary to determine the best strategy. In Europe, ViV TAVR was first CE Mark approved in 2013 and FDA approved for inoperable and high-risk patients (30-day surgical mortality >8% by STS PROM) with the balloon expandable valves [25] and subsequently with self-expandable valves in 2015 [26]. One of the limitations of ViV TAVR is the risk of severe PPM, since the transcatheter valve sits within the surgical valve's true ID. This is especially true in smaller surgical valves and has been associated with higher one-year mortality in the Valve-in-Valve International Data Registry; surgical valves ≤21 mm had significantly higher one-year mortality (25%) compared to valves ≥23 and ≤25 mm (18%), and valves ≥27 mm (7%) (*p* = 0.001) [27]. Surgical prosthesis ≤21 mm (HR = 2.04, [95% CI: 1.14, 3.67], *p* = 0.02) and stenosis as the primary mechanism of failure (vs. regurgitation; HR = 3.07, (95% CI: 1.33, 7.08), *p* = 0.008) were significant risk factors for one-year mortality [27]. To improve the hemodynamic results of ViV TAVR, different techniques can be employed, including implanting the transcatheter valve high within the surgical valve, utilizing a supra-annular transcatheter valve, and bioprosthetic valve fracture (BVF) and bioprosthetic valve remodeling (BVR).

### 3.1. Bioprosthetic Valve Fracture and Bioprosthetic Valve Remodeling

The bioprosthetic valve fracture was first described by Nielsen-Kudsk et al. in 2015. In small mitroflow bioprostheses, a high-pressure balloon predilatation with an ATLAS Gold balloon fractured the annular stent ring of the SAVR valves and a 20 mm SAPIEN XT was placed in the 19 mm Mitroflow and a SAPIEN 3 23 mm TAVR valve was placed in a 21 mm mitroflow without any complications [28]. The BVF allows for greater expansion of the transcatheter valve and the implantation of a larger, more fully expanded, transcatheter valve. However, BVF is not an option for all patients. Bioprosthetic valve remodeling (BVR) is similar in concept with the intention of fully expanding the TAVR without fracturing the surgical valve annulus. Although BVR can improve the gradient across the valve and leaflet coaptation of the more fully expanded TAVR leaflets, the annulus diameter is always constrained by the initial surgical valve platform.

#### 3.1.1. Preoperative Assessment

For BVF/BVR, the implanted surgical valve is first identified to determine if it can be fractured or remodeled. Valve fracture is an option for some bioprosthetic valves including Magna, Magna Ease, Perimount 2800, Mitroflow, Mosaic, and Bicor Epic (Table 2) while valve remodeling/stretching is an option for Trifecta, Carpentier-Edwards standard and supraannular, Inspiris, and Perimount 2700 [29] (Table 3). The Medtronic Hancock II and Medtronic Avalus valves cannot be fractured or remodeled [29].

#### 3.1.2. Procedure

The BVF fractures the internal annular ring within the sewing cuff of the surgical valve to allow for maximal expansion of the new valve and results in improved hemodynamics post-ViV TAVR deployment. Following initiation of rapid ventricular pacing, a noncompliant balloon is rapidly filled with dilute contrast and pressurized using an indeflator until fracture occurs [29]. Specific valves fracture at different pressures (Table 2). While fracture can be difficult to confirm, the best indicator is an acute drop in the indeflator pressure near the fracture threshold for the given surgical valve and a vibration or shutter felt through the shaft of the noncompliant balloon [29]. Optimal balloon size should be determined by the ID of the surgical valve, the transcatheter valve used, the anticipated increase in diameter following fracture, the aortic root and left ventricular outflow tract (LVOT) anatomy, and the location of the coronary arteries [29]. A multicenter study by Allen KB et al. found the best hemodynamic result was achieved when BVF was performed after ViV TAVR and with a balloon at least 3 mm larger than the true internal diameter of the surgical valve [30].

#### 3.1.3. Procedural Planning

In a native aortic valve, the ID is measured at the level of the aortic annulus and used to determine the size of the transcatheter valve to be implanted. For ViV TAVR, the size of the *in-situ* valve, particularly the ID, determines the largest transcatheter valve that can be implanted. In both cases, a degree of oversizing is chosen to ensure secure fixation. The ID of the *in-situ* surgical valve can be obtained from the manufacturer; however, the true internal diameter is affected by how the leaflets are secured (internal vs. external); internally mounted leaflets can reduce the true ID by at least 2 mm [31]. The Valve in Valve application (https://apps.apple.com/us/app/valve-in-valve/id655683780) [32] is a useful resource for additional details in selecting the appropriate valve for ViV TAVR. In addition, surgical valve leaflet height should be taken into consideration if implanting a Sapien valve to prevent leaflet overhang.

**Figure 2:**
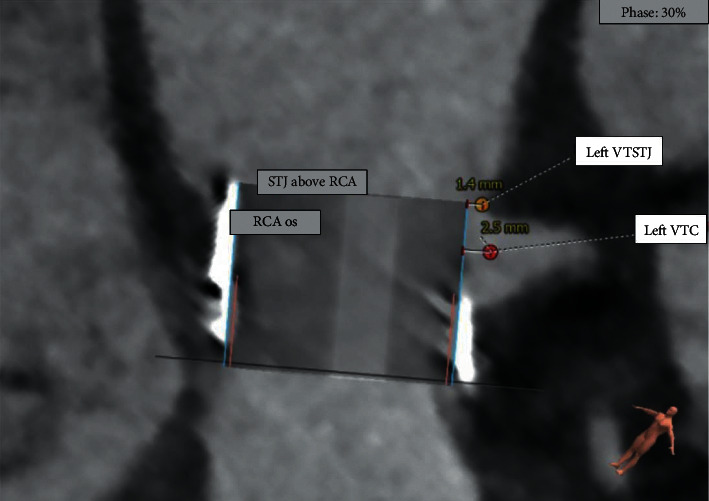
Procedural preplanning with 3D Reconstruction and virtual valve in a failed 21 mm Magna surgical valve. With the smallest sized balloon expandable valve, 20 mm, the valve to coronary (VTC) distance to the left main coronary ostium (2.5 mm) and valve to sinotubluar junction(VTSTJ) (1.4 mm) are not adequate.

Cardiac gated multidetector computed tomography (MDCT) is used to determine the inner diameter and area of the failed valve for selection of the appropriate TAVR. Under-sizing can result in paravalvular regurgitation and embolization, but oversizing may result in incomplete expansion, increased gradients, and coronary obstruction [31, 33]. During ViV TAVR, coronary obstruction is more common than in native TAVR due to the supra-annular implantation of most surgical valves. Preoperative CTA is used to predict the risk of coronary occlusion as the SAVR leaflets are pushed toward the coronary ostia during ViV TAVR and create a complete tube of leaflet tissue that can reach the level of the sinotubular junction (STJ). Coronary height, the distance from the coronary ostium to the aortic valve annulus, is one of the important factors to consider when evaluating risk for coronary obstruction [34]. Lower coronary heights are more often seen in patients with *in-situ* surgical valves compared to those with native valves. Therefore, in ViV TAVR planning, the coronary height should be measured from the sewing ring of the basal plane of the prosthesis and not the true native annular plane [35]. On preoperative CTA analysis, crucial factors include identification of the failed leaflets, bioprosthesis angulation in relation to the aortic annulus, coronary ostia height, sinus of Valsalva diameter, STJ height, and SAVR leaflet length. The distance from the surgical valve leaflet to the coronary ostia, the valve to coronary (VTC) distance, predicts the feasibility of ViV TAVR and the risk of coronary obstruction. A VTC of 4 mm or greater is necessary for ViV TAVR (Figure 2). Stentless bioprosthetic valves and stented bioprosthetic valves with externally mounted leaflets have an increased risk of coronary obstruction. Those at highest risk for coronary obstruction are female patients, coronary ostial height <10 mm, sinus of Valsalva (SOV) diameter <30 mm, VTC distance <4 mm, and previous aortic bioprostheses, particularly those with stented valves with externally mounted leaflets or stentless surgical valves (OR 7.67, [95% CI: 3.14, 18.7], *p* < 0.0001) [36]. When BVF is performed, the gain in annular dimension is 3-4 mm; therefore, the VTC distance should be at least 5 mm in order to accommodate valve expansion [37]. Additionally, when evaluating for BVF, the SOV diameter and STJ height must be measured to ensure the sinus is large enough to accommodate the increased valve size without root rupture or sinus sequestration and the STJ height is adequate to accommodate full leaflet excursion without the leaflet reaching the level of the STJ (minimum valve to STJ distance of 2 mm is suggested) [38].

BVF results in an increase in the ID of the surgical valve of 3-4 mm and the selection of transcatheter valve size should be based on this anticipated increased ID. BVF can be performed before or after ViV TAVR. When performed before ViV TAVR, it effectively fractures the surgical valve but does not ensure adequate expansion of the subsequent TAVR. If BVF is performed after ViV TAVR, it fractures the surgical valve and fully expands the transcatheter valve. BVF has been shown as a beneficial strategy to prevent PPM, in particular for small surgical prostheses. Despite patients with larger surgical valves having a lower risk of PPM and high gradients, BVF can still be utilized to promote full transcatheter valve expansion. However, BVF remains understudied in patients with larger surgical valve sizes [39]. BVF results in reduced transvalvular gradients and increased EOA; for optimal results, it is suggested that BVF be performed after ViV TAVR and with a non-compliant balloon at least 3 mm larger than the true ID of the surgical valve being fractured [30] but with a balloon no larger than the waste of the self-expanding valve to avoid damage to the valve leaflets.

#### 3.1.4. Adjunctive Techniques

During SAVR, commissure-to-commissure alignment is maintained, while in TAVR the orientation of the commissures is often random and not consistently achievable. Tang et al. found that the Evolut “hat” marker and the ACURATE-neo commissural post facilitated improved commissural alignment and reduced coronary artery overlap [40]. The crimping of the Sapien 3 valve had no impact on commissural alignment predictability in the study. Commissural alignment facilitates coronary access and future options for transcatheter management if the ViV TAVR fails. This is an area of active investigation as younger and lower-risk patients, with a long life expectancy, receive ViV TAVR.

Bioprosthetic or native Aortic Scallop Intentional Laceration to prevent Iatrogenic Coronary Artery Obstruction (BASILICA) is an electrosurgical leaflet modification technique which is effective in preventing coronary obstruction in native and bioprosthetic valves. In patients at high risk of coronary obstruction due to a VTC distance <4 mm, or at risk of sinus sequestration due to a narrow SOV, short STJ height, and/or long bioprosthetic valve leaflets, using an electrified wire, the nadir of the bioprosthetic leaflet is crossed and leaflet lacerated to create a *V* shaped opening (leaflet splay) to increase blood flow and access to the coronary artery at risk. In a series of 30 patients in the initial BASILICA feasibility study, freedom from coronary obstruction was 95% and no patient required reintervention [41]. Patients in whom BASILICA is predicted to result in inadequate “splay” (particularly problematic for failed TAVR valves and the feasibility of TAVR-in-TAVR), balloon-assisted BASILICA can be utilized to expand the traversal point outward by balloon inflation prior to laceration [42].

### 3.2. Surgical Techniques

In addition to ViV TAVR, redo-SAVR is another option. Although redo-SAVR has traditionally been considered a higher-risk operation compared to primary AVR, the mortality of redo SAVR is 1–5% with appropriate patient selection [43, 44]. Comparing ViV TAVR and redo-SAVR in a single center series, those undergoing ViV TAVR were older and had more comorbidities including peripheral arterial disease, congestive heart failure with NYHA class III or IV symptoms, hypertension, prior myocardial infarction (MI), and history of atrial fibrillation; however, postoperative outcomes were similar. The ViV-TAVR group had shorter lengths of stay while the redo-SAVR group had improved hemodynamics [44]. In a meta-analysis comparing ViV TAVR and redo-SAVR with degenerated bioprosthetic valves, all cause 30-day mortality was higher in the redo-SAVR group and there was no significant difference in stroke, MI, or permanent pacemaker at mid-term follow-up of up to 5 years. However, ViV TAVR was associated with a higher risk of PPM and greater transvalvular pressure gradients postimplantation [45]. Both ViV TAVR and redo-SAVR are viable options and patient selection is key to success; higher-risk patients and patients with larger valves benefit more from ViV TAVR while younger patients and patients with smaller valves benefit more from redo-SAVR.

## 4. Conclusion

Due to the increase in bioprosthetic valve utilization for the treatment of aortic valve disease and patients with longer life expectancy, bioprosthetic valve failure is becoming a significant problem requiring innovative treatment strategies. Redo-SAVR has traditionally been the only treatment modality for failed biologic valves, but many elderly patients are not candidates for a second operation or do not wish to undergo a redo-sternotomy. Valve fracture provides one strategy to achieve optimal hemodynamics by increasing the size of the annulus for ViV TAVR. BVF is especially useful in patients with small surgical valves to decrease the risk of PPM by removing the constraints of placing a transcatheter valve within a rigid surgical bioprostheses and when performed after ViV-TAVR facilitates expansion of the transcatheter valve. Although in the US ViV TAVR is reserved for high-risk patients, risk drift is expected with this technology. Not only do we need to provide a solution to the initial failed surgical valve, but planning for a third valve when the VIV TAVR fails must be considered in the lifetime management of aortic valve disease. It may be that all patients, not just those with small annuli, benefit long-term from valve fracture and additional study is needed.

## Figures and Tables

**Table 1:** Risk /protective factors for SVD.

	Hazard ratio (95% confidence interval)	*p* value
Risk factors		
Younger age		
Age per 1 year decrease	1.10 (1.06, 1.12)	<0.0001
Increasing BSA	1.77 (1.04, 3.01)	0.034
PPM	1.95 (1.56, 2.43)	<0.001
Smoking	2.28 (1.37, 3.79)	0.0015

*Protective factor*		
Anticalcification preparation	0.41 (0.19, 0.89)	0.025
Older age		
Age >60 years	0.12 (0.06, 0.23]	<0.0001
Age >65 years	0.06 (0.02, 0.21)	<0.0001
Age >70 years	0.06 (0.01, 0.28)	0.0004

BSA = body surface area; PPM = patient prosthesis mismatch. Adapted from Ochi A, Cheng K, Zhao B, Hardikar AA, Negishi K. Patient Risk Factors for Bioprosthetic Aortic Valve Degeneration: A Systematic Review and Meta-Analysis. *Heart Lung Circ.* 2020; 29 : 668–678.

**Table 2:** Surgical prosthesis amenable to valve fracture.

	Make	Stented/Stentless	Leaflets	Fracture threshold (atm)	Valve sizes	ID (mm)	Profile height (mm)
CE magna	Edwards lifesciences	Stented	Internal	22–24			
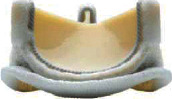					19	18.0	14.0
					21	20.0	15.0
					23	22.0	16.0
					25	24.0	17.0
					27	26.0	18.0
					29	28.0	19.0

CE magna ease	Edwards lifesciences	Stented	Internal	18			
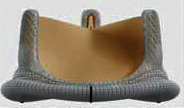					19	18.0	13.0
					21	20.0	14.0
					23	22.0	15.0
					25	24.0	16.0
					27	26.0	17.0
					29	28.0	18.0

Perimount 2800/2900	Edwards lifesciences	Stented	Internal	20			
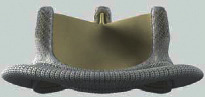					19	18.0	14.0
					21	20.0	15.0
					23	22.0	16.0
					25	24.0	17.0
					27	26.0	18.0
					29	28.0	19.0

Mitroflow	Sorin group	Stented	External	12			
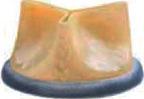					19	15.4	11.0
					21	17.3	13.0
					23	19.0	14.0
					25	21.0	15.0
					27	22.9	16.0

Mosaic	Medtronic	Stented	Internal	10^*∗*^			
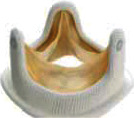					19	17.5	13.5
					21	18.5	15.0
					23	20.5	16.0
					25	22.5	17.5
					27	24.0	18.5
					29	26.0	20.0

Epic	Abbott	Stented	Internal	8			
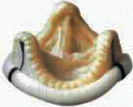					19	18.7	14.0
					21	20.8	15.0
					23	22.6	16.0
					25	24.5	17.0
					27	26.3	19.0

^*∗*^The Mosaic valve has been manufactured with two different materials and behaves differently during BVF depending on the material used to manufacture the frame. If the frame is made of Derlin, fracture occurs ∼10–12 atm. If comprised of the high-performance thermoplastic polyetheretherketone (PEEK) (a small amount in the Mosaic valve) it cannot be fractured but can me remodeled; continue to increase the inflation device pressure beyond 12 atm and at about 18 atm, the valve frame will begin to stretch. Inflate to ∼22 atm to achieve maximal expansion. Allen KB, Chhatriwalla AK, Saxon JT, et al. Bioprosthetic valve fracture: Technical insights from a multicenter study. *J Thorac Cardiovasc Surg.* 2019; 158 (5):1317–1328 e1311.

**Table 3:** Surgical prosthesis amenable to valve remodeling.

	Make	Stented/Stentless	Leaflets	Valve sizes	ID (mm)	Profile height (mm)
Trifecta	Abbott	Stented	External			
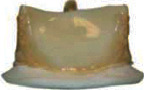				19	17	15
				21	19	16
				23	21	17
				25	23	18
				27	25	19

CE standard porcine	Edwards lifesciences	Stented	Internal			
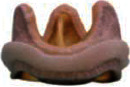				19	17	15
				21	19	16
				23	21	16
				25	23	18
				27	25	18
				29	27	19
				31	29	19

CE supra-annular	Edwards lifesciences	Stented	Internal			
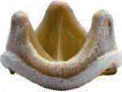				21	19	15
				23	21	16
				25	23	17
				27	25	17

Inspiris resilia	Edwards lifesciences	Stented	Internal			
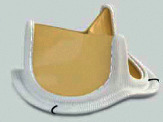 0				19	18	13
				21	20	14
				23	22	15
				25	24	16
				27	26	17
				29	28	19

Perimount 2700	Edwards lifesciences	Stented	Internal			
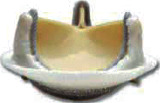 1				19	18	13
				21	20	14
				23	22	15
				25	24	16
				27	26	17
				29	28	18

## Data Availability

This is a review article; data can be found on Pubmed as per the list of references.

## Abbreviations

AVR: Aortic valve replacement
BASILICA: Bioprosthetic or native aortic scallop intentional laceration to prevent iatrogenic coronary artery obstruction
BSA: Body surface area
BVD: Bioprosthetic valve dysfunction
BVF: Bioprosthetic valve fracture
BVR: Bioprosthetic valve remodeling
CE: Conformitè europèenne
CTA: Computed tomography angiography
EOA: Effective orifice area
FDA: Food and drug administration
HR: Hazard ratio
ID: Internal diameter
LVOT: Left ventricular outflow tract
MDCT: Multidetector computed tomography
MI: Myocardial infarction
NSVD: Nonstructural valve deterioration
NYHA: New York heart association
PPM: Patient-prosthesis mismatch
SAVR: Surgical aortic valve replacement
SVD: structural valve deterioration
TAVR: Transcatheter aortic valve replacement
US: United states
VARC-3: Valve academic research consortium 3
ViV: Valve-in-valve.
